# Retrospective and comparative assessment of MDRD, CKD-EPI, and EKFC equations for estimating glomerular filtration rate in a clinically heterogeneous hospital population

**DOI:** 10.1007/s11255-026-05288-w

**Published:** 2026-07-24

**Authors:** Matteo Baldetti, Riccardo Belardi, Silvia Velocci, Riccardo Luffarelli, Stefano Di Carlo, Sergio Bernardini, Alessandro Terrinoni, Marilena Minieri

**Affiliations:** 1 Department of Experimental Medicine, University of Rome Tor Vergata, Via Montpellier 1, 00133 Rome, Italy; 2 Unit of Laboratory Medicine, University Hospital of Rome Tor Vergata, Viale Oxford, 81, 00133 Rome, Italy

**Keywords:** eGFR, EKFC, MDRD-IDMS, CKD-EPI 2009, KDIGO

## Abstract

**Background:**

Chronic kidney disease (CKD) is projected to become the fifth leading cause of death by 2040. Accurate estimation of glomerular filtration rate (eGFR) is essential for diagnosis, staging, and management. Our laboratory still uses Modification of Diet in Renal Disease (MDRD) traceable to the Isotope Dilution Mass Spectrometry (IDMS) equation, which is now considered outdated due to reduced accuracy at higher eGFR values. More recent equations may offer improved performance. This study aimed to compare these four eGFR equations from both analytical and clinical perspectives to support the change of equation used in our laboratory.

**Methods:**

Retrospectively, this study compared the routinely used MDRD equation with alternative eGFR formulas in order to evaluate the impact of adopting newer equations.

**Results:**

The study population included 1438 patients (886 males and 552 females; mean ages of 60 and 63 years for males and females, respectively). MDRD-IDMS showed limited consistency above 80 mL/min/1.73 m^2^. Chronic Kidney Disease Epidemiology Collaboration (CKD-EPI) 2009 had the strongest agreement and similar staging, while CKD-EPI 2021 generated higher eGFR values. European Kidney Function Consortium (EKFC) reclassified about 12% of patients into more advanced stages, possibly influencing clinical management.

**Conclusions:**

Both CKD-EPI 2009 and EKFC proved to be suitable alternatives to MDRD-IDMS. However, CKD-EPI 2009 was identified as the most potentially appropriate equation for implementation in our hospital setting due to the characteristics of the served population and its balanced impact on CKD stage reclassification.

**Supplementary Information:**

The online version contains supplementary material available at https://doi.org/10.1007/s11255-026-05288-w.

## Introduction

Chronic kidney disease (CKD) is defined as eGFR < 60 mL/min/1.73 m^2^ or markers of kidney damage (e.g., ACR ≥ 30 mg/g, structural abnormalities, persistent hematuria) lasting for more than 3 months. Although the epidemiological data are not entirely consistent, as they are largely derived from cohort studies with heterogeneous populations, the global prevalence of CKD is estimated at around 11%, with diabetes and hypertension as leading causes in high- and middle-income countries [1]. Projections suggest that by 2040, CKD will rank as the fifth leading cause of death worldwide [2]. From a therapeutic perspective, kidney-preserving treatment represents a valuable option before dialysis or kidney replacement becomes necessary. Its primary goal is to slow disease progression and improve overall quality of life by addressing both CKD-related and non-CKD-related symptoms [3]. The most widely used biomarker for eGFR calculation is serum creatinine, even though it shows a few limitations [4]. Serum creatinine–eGFR relationship is hyperbolic and creatinine also depends on non-renal factors, especially muscle mass, which varies by sex, age, and body weight [5]. To reduce creatinine’s dependency on muscle mass and improve renal function estimation, cystatin C has been introduced as an alternative biomarker, which helped eliminating the need for timed urine collection [6]. Evidence supports its utility for earlier CKD detection and better risk stratification [7]. After the Cockroft–Gault being the first widely used equation for GFR estimation [5], the Modification of Diet in Renal Disease (MDRD) study developed seven equations using stepwise regression, incorporating variables, such as age, sex, race, serum and urine urea, albumin, and creatinine from 1070 of 1628 patients. These equations were validated against measured GFR and Eq. 7 proved most accurate, estimating GFR directly from serum creatinine and eliminating the need for 24 h urine collection. Initially, creatinine was measured using a non-standardized assay, which was later recalibrated to produce values traceable to the Isotope Dilution Mass Spectrometry (IDMS) reference method, enabling comparable eGFR results and more accurate measurements [7]. Despite its widespread adoption, the MDRD equation tends to underestimate GFR and overestimate CKD prevalence in the general population [4], also extremely overestimating eGFR when serum creatinine levels are low [8]. Later on, new equations were developed, validated, and proved to be differentially more adequate than MDRD-IDMS. For instance, Chronic Kidney Disease Epidemiology Collaboration (CKD-EPI) 2009 was found to be as accurate as MDRD for eGFR values below 60 mL/min/1.73 m^2^, while providing greater accuracy and lower bias at higher eGFR values. Moreover, this equation provides a better classification of patients into the different CKD stages [9]. In 2021, the same research group developed a refined equation excluding race to remove this as a factor, aiming to improve CKD detection in Black individuals. However, it underestimates GFR in Black individuals and overestimates it in non-Black populations, impacting CKD epidemiology and management [10]. Evidence from European cohorts shows reduced performance in non-Black populations, and the Italian Society of Clinical Biochemistry and Clinical Molecular Biology (SiBioC) advised against its use [11]. One of the latest equations for GFR estimation is the European Kidney Function Consortium (EKFC) equation. This aimed to improve GFR estimation across the entire age spectrum, measuring urinary clearance of exogenous tracers [12]. With regard to CKD staging, when compared with CKD-EPI 2009 in a large northern European cohort, the EKFC equation reclassified most participants into lower eGFR categories, increasing CKD prevalence and potentially impacting clinical decisions [13]. Our laboratory is still using the MDRD-IDMS equation, which is now deemed outdated and is no longer mentioned in the latest CKD management guidelines. For this reason, we are considering switching to a newer equation. Currently, our laboratory does not have the resources to directly measure GFR and assess the accuracy of different estimating equations. Therefore, this study was designed as a preliminary evaluation of MDRD-IDMS, CKD-EPI 2009, CKD-EPI 2021, and EKFC, focusing on differences in eGFR estimation and CKD staging to inform the potential replacement of MDRD-IDMS in routine clinical practice.

## Materials and methods

### Study design and data description

This retrospective study included adult patients (*n* = 1438; ≥ 18 years) with serum creatinine measurements collected during routine care at the University Hospital of Rome Tor Vergata between 2015 and 2022. The initial dataset of over 3000 records was used to determine AKI and CKD prevalence. Duplicates were then removed by retaining one entry per patient, resulting in 1438 unique cases. Ethics committee approval was not required due to the retrospective design.

### MDRD-IDMS, CKD-EPI, EKFC equations

We estimated GFR using creatinine-based equations. The formulas applied were the MDRD-IDMS, CKD-EPI 2009, CKD-EPI 2021, and EKFC 2021. Further comparisons between combined creatinine–cystatin C versions of CKD-EPI and EKFC were performed, but we decided not to include them in the main paper as these versions are only used when encountering specific clinical scenarios in which they can be more suitable than creatinine-based eGFR.

### Statistical analysis

#### Correlation between equations

First, data normality was assessed by visual inspection of Q–Q plots as well as by calculating kurtosis and skewness. The overall agreement between two equations was evaluated using the Passing–Bablok method and Bland–Altman plots. Spearman’s correlation coefficient was then calculated to assess the linear association between the values derived from different equations.

#### CKD stage classification

CKD staging was evaluated for each equation by cross-tabulating the proportion of individuals with eGFR values within specific ranges (≥ 90, 60–89, 45–59, 30–44, 15–29, < 15 mL/min/1.73 m^2^), corresponding to the KDIGO 2024 Clinical Practice Guideline for the Evaluation and Management of Chronic Kidney Disease classification scheme (G1, G2, G3a, G3b, G4, G5, respectively) [14]. Inter-rater agreement was assessed using Cohen’s kappa coefficient and the overall percentage agreement.

#### Statistical software

All statistical tests were performed with R Studio (R Core Team (2024). _R: A Language and Environment for Statistical Computing_. R Foundation for Statistical Computing, Vienna, Austria. < https://www.R-project.org/ > .).

#### Creatinine and determination methods

From 2015 to 2017, creatinine levels were measured using the Dimension Vista® System with the kinetic Jaffe method. From 2017 to 2022, creatinine was quantitatively assessed using an enzymatic method on the ARCHITECT cSystems platform from Abbott. This method involves the hydrolysis of creatinine to creatine, and its conversion to sarcosine and urea. No calibration study or method comparisons was available. Spearman's correlation, Bland–Altman analysis, and CKD stage comparison were performed between the two time periods during which different serum creatinine measurement methods were used. The results of these analyses are included in the supplementary material.

## Results

### Study population characteristics

We first performed a descriptive analysis of the retrospective cohort. Table 1 summarizes the characteristics of 1438 patients (886 males, 61.6%; 552 females, 38.4%) with a median age of 64 years (IQR 51–74). Most patients were aged 40–79, followed by those ≥ 70, while fewer were 18–39. Median serum creatinine was 1.05 mg/dL in men and 0.80 mg/dL in women; median cystatin C was 1.17 mg/L and 1.18 mg/L, respectively. Overall medians were 0.95 mg/dL (Scr) and 1.19 mg/L (Scy). Race/ethnicity data were not systematically recorded in the institutional laboratory database and were therefore not available for analysis. Most patients were from the ICU (*n* = 754, followed by external referrals (*n* = 364), with the remainder from other departments.

**Table 1:** Demographic characteristics and descriptive statistics of the population (*N* = 1438)

Demographics	
Age, years	64 (74–51)

	Males	Females	Total
N. patients	886 (61,6%)	552 (38,4%)	*N* = 1438
By age			
18–39 years	100	41	141 (9.8%)
40–69 years	492	287	779 (54.17%)
≥ 70 years	294	224	518 (36.02%)
Median Scr	1,05 mg/dL	0,8 mg/dL	0,95 mg/dL
Median Scy	1,17 mg/L	1,18 mg/L	1,19 mg/L

Department	# Patients
Intensive Care Unit (ICU)	754
External patients	364
Surgery and transplants	137
Cardiac surgery	42
Internal Medicine	40
Gastroenterology	27
Hepatology	17
Hypertension and Nephrology	13
Infectious disease	15
Stem cells transplant department	11
Cardiothoracic anesthesia	9
Endocrinology and diabetology	9

61.6% of the population which was matter of the research was composed of males, while 38.4% of females. This table also shows the different departments in which the patients were hospitalized and the number of external patients

Furthermore, we performed additional analyses on our longitudinal data to estimate AKI prevalence within the cohort. We identified 100 patients (out of 1438) meeting the AKI criteria based on a serum creatinine increase (delta) [15]. Interestingly, all these patients were admitted to the ICU. As discussed, we cannot definitively determine whether these fluctuations reflect actual deterioration of renal function or hemodynamic factors, such as intensive fluid resuscitation and muscle mass loss, which are particularly prevalent in this critical setting.

On the other hand, CKD prevalence was defined as an estimated glomerular filtration rate (eGFR) below 60 mL/min/1.73 m^2^ persisting for at least three months. CKD was identified in 37 patients, of whom 26 were outpatients, while the remaining patients originated from other departments. Cohen’s kappa concordance was calculated for CKD staging on this small CKD cohort. The highest level of agreement (κ = 0.94) was observed between CKD-EPI 2009 and EKFC. This was followed by CKD-EPI 2009 versus CKD-EPI 2021 (*κ* = 0.91), while the lowest agreement was found between MDRD and CKD-EPI 2009 (*κ* = 0.75).

### Analytical comparison of eGFR equations

**Fig. 1:**
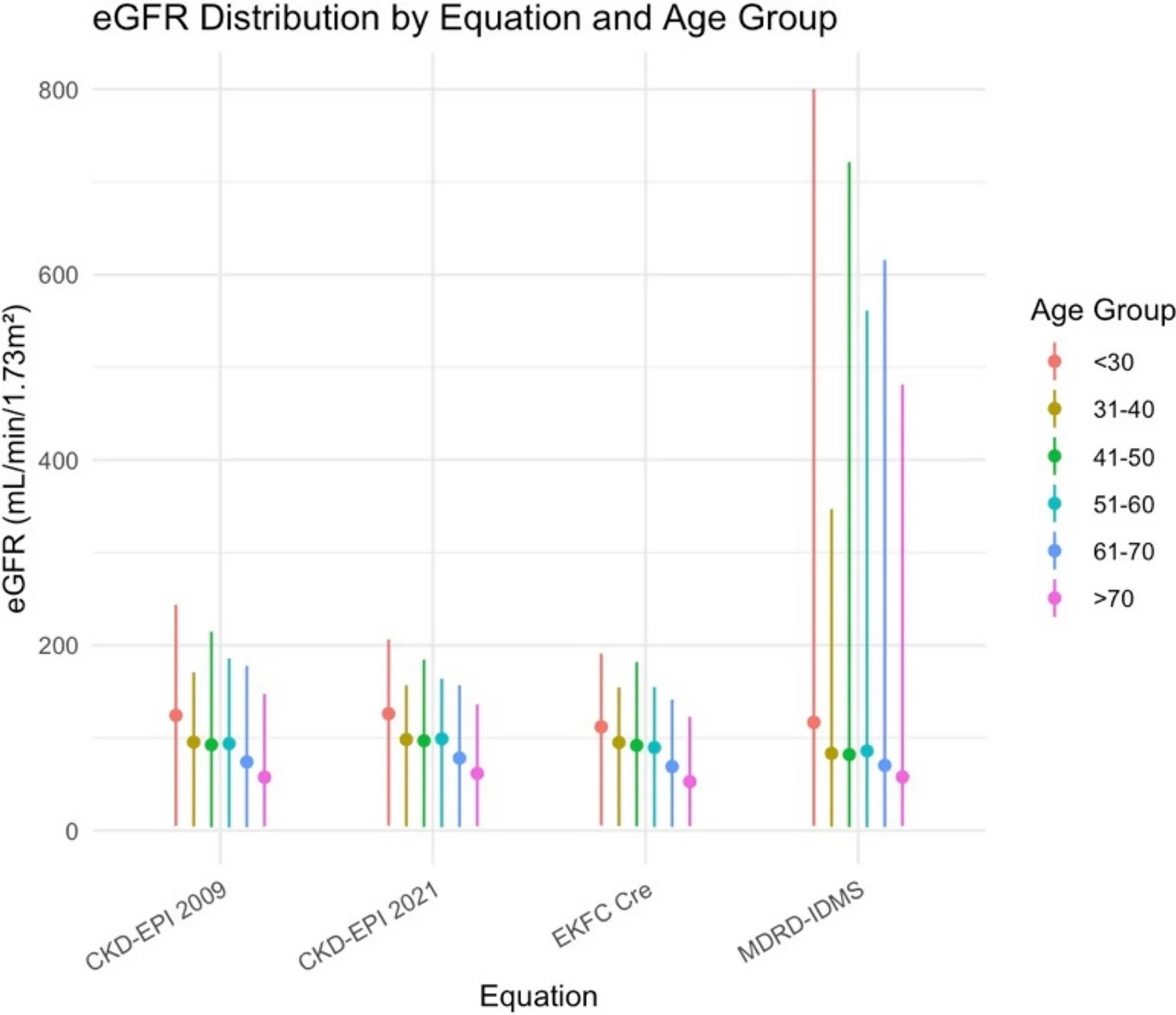
eGFR by CKD-EPI 2009, CKD-EPI 2021, EKFC, and MDRD-IDMS, stratified by age (< 30 to > 70). Values are medians with dispersion; eGFR declines with age across all equations

We analyzed eGFR distributions by age range (Fig. 1). MDRD-IDMS shows the greatest variability and a wider distribution, while eGFR medians decline progressively with age.

MDRD-IDMS has the highest IQR (64.8) and SD (68.2), reflecting scattered values, especially at high eGFR. CKD-EPI 2009 shows lower dispersion (SD 59.22; IQR 37.65) and a more consistent distribution. Analytically, we started by comparing MDRD-IDMS with the CKD-EPI 2009.

**Fig. 2:**
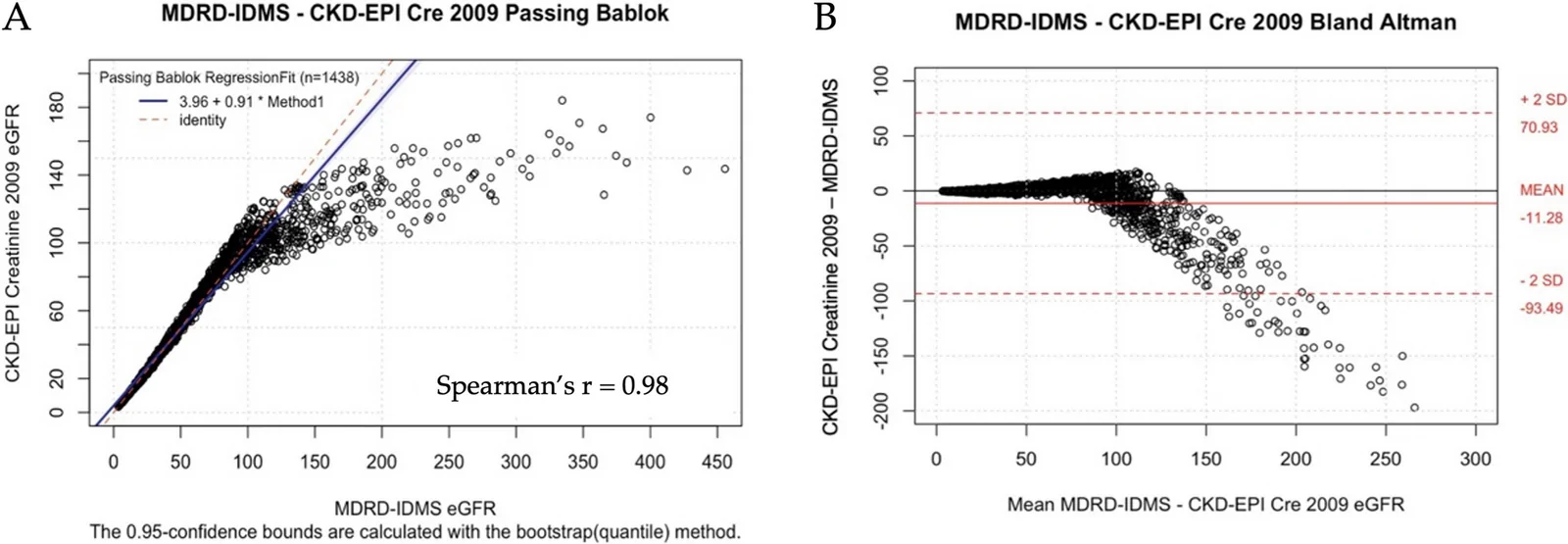
Passing–Bablok and Bland–Altman analysis between MDRD-IDMS and CKD-EPI 2009. **A** Passing–Bablok regression shows deviation from linearity at higher eGFR values, while the **B** Bland–Altman plot illustrates a negative bias increasing with higher GFR levels

Figure 2, Panel A shows very high correlation with CKD-EPI 2009 (*r* = 0.98) though Passing–Bablok regression and Bland–Altman analyses (Fig. 2, Panel B) reveal nonlinearity above approximately 120 mL/min/1.73 m^2^, where MDRD-IDMS tends to provide with disproportionately higher eGFR values than CKD-EPI 2009. Overall, the mean value in the Bland–Altman analysis (− 11.26) confirms that MDRD-IDMS provides higher eGFR values than CKD-EPI 2009 throughout the analyzed eGFR range. However, this difference could be attributed to the high number of eGFR values above approximately 80 mL/min/1.73 m^2^.

**Fig. 3:**
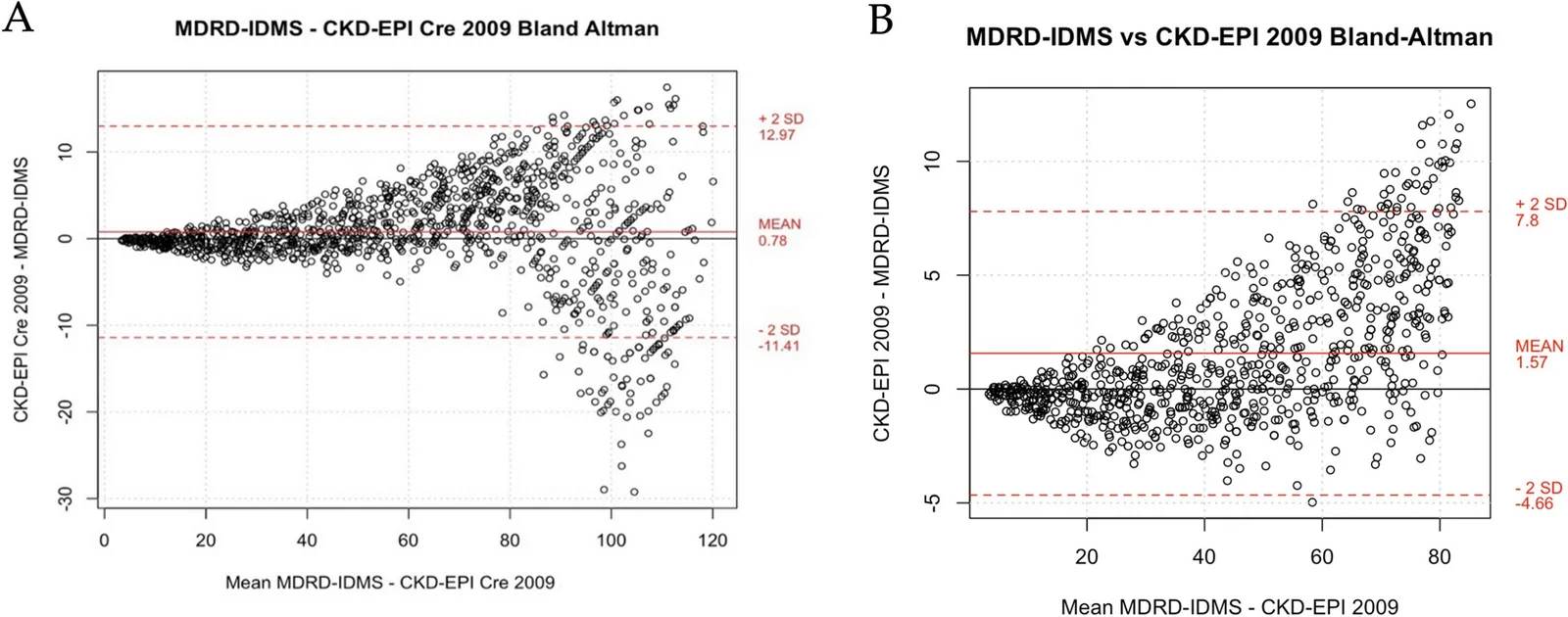
Bland–Altman analysis between CKD-EPI 2009 and MDRD-IDMS. **A** Estimated GFR values lower than 120 mL/min/1.73 m^2^ only were included in the analysis to exclude any potential bias caused by MDRD-IDMS with high eGFR values. **B** A further filtering of eGFR values (lower than 80 mL/min/1.73 m^2^) was performed to reduce bias introduced by MDRD-IDMS

To reduce the bias introduced by the estimates from MDRD-IDMS above the aforementioned range, and better visualize the virtual loss linearity, we further limited the analysis to eGFR (MDRD-IDMS) ≤ 120 and 80 mL/min/1.73 m^2^ (Fig. 3).

Restricting the analysis to ≤ 120 mL/min/1.73 m^2^ (Fig. 3A), the Bland–Altman mean shifted from − 11.28 to 0.78, indicating higher eGFR values with CKD-EPI 2009. Further restriction to < 80 mL/min/1.73 m^2^ (Fig. 3B) reduced the mean further, confirming that CKD-EPI 2009 yields progressively higher eGFR values than MDRD-IDMS in the clinically relevant range. Subsequently, we analyzed the differences in CKD staging between the two equations. The cross-tabulation results are summarized in Table 2.

**Table 2:**
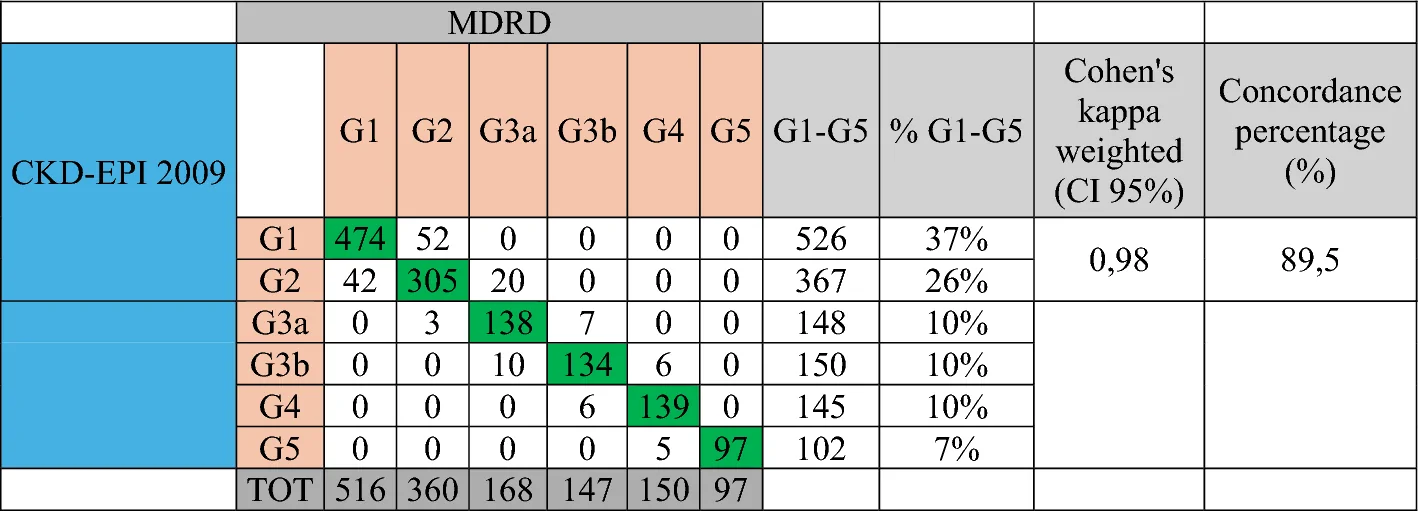
Cross-tabulation results of MDRD-IDMS and CKD-EPI 2009 comparison

This table shows a balanced reclassified toward more advanced and earlier CKD stages overall.

**Fig. 4:**
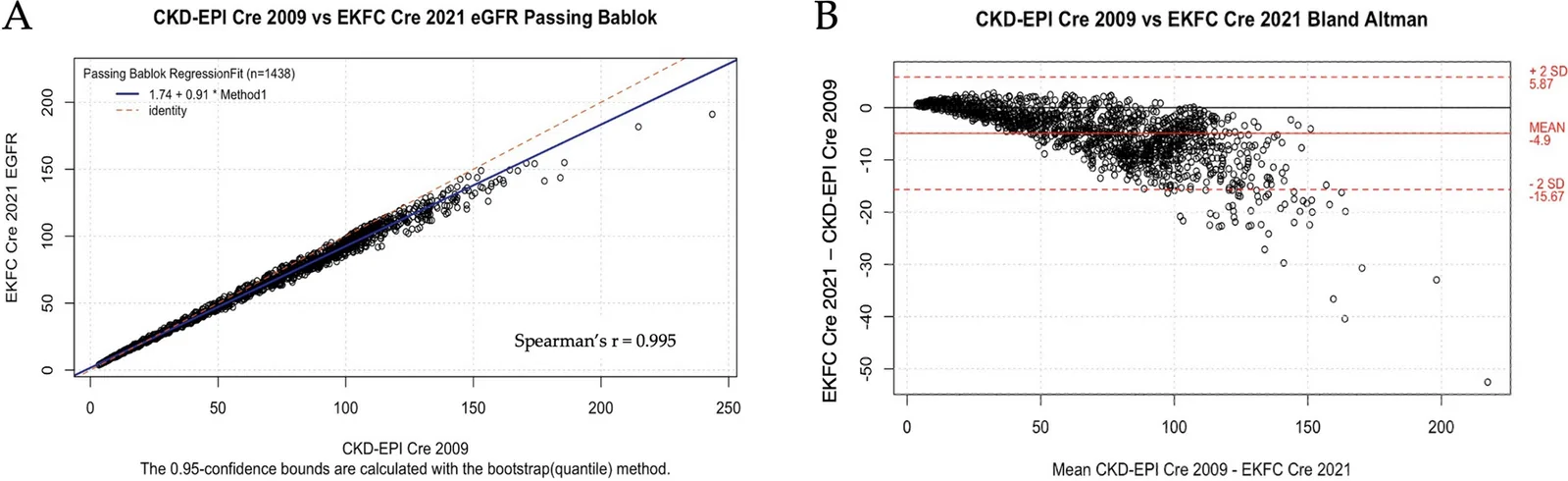
Analytical comparison between CKD-EPI 2009 and EKFC 2021. **A** Passing–Bablok analysis shows that the equations are highly correlated when it comes to estimating eGFR. A small proportional error is reported by the Eqs. (1.74). **B** Bland–Altman analysis shows a mean of − 4.9, indicating the EKFC tendency to progressively provide lower eGFR values than CKD-EPI 2009

Our results show high concordance between MDRD-IDMS and CKD-EPI 2009 for CKD staging. CKD-EPI 2009 reclassified 10.5% of patients, mainly in early stages: 42 from G1 to G2, 52 from G2 to G1 (3 to G3a), and 30 of 168 G3a to adjacent stages. Overall, 85 patients were reclassified to earlier stages and 66 to more advanced ones. We then compared CKD-EPI 2009 with EKFC 2021. Figure 4 shows excellent correlation (*r* = 0.995), while Bland–Altman analysis indicates that EKFC yields progressively lower eGFR values than CKD-EPI 2009.

Table 3 shows that EKFC reclassified 174 patients into more advanced CKD stages, particularly from G1 to G2 and G2 to G3a. In spite of this fact, Cohen’s kappa shows a very high concordance coefficient (0.97).

**Table 3:**
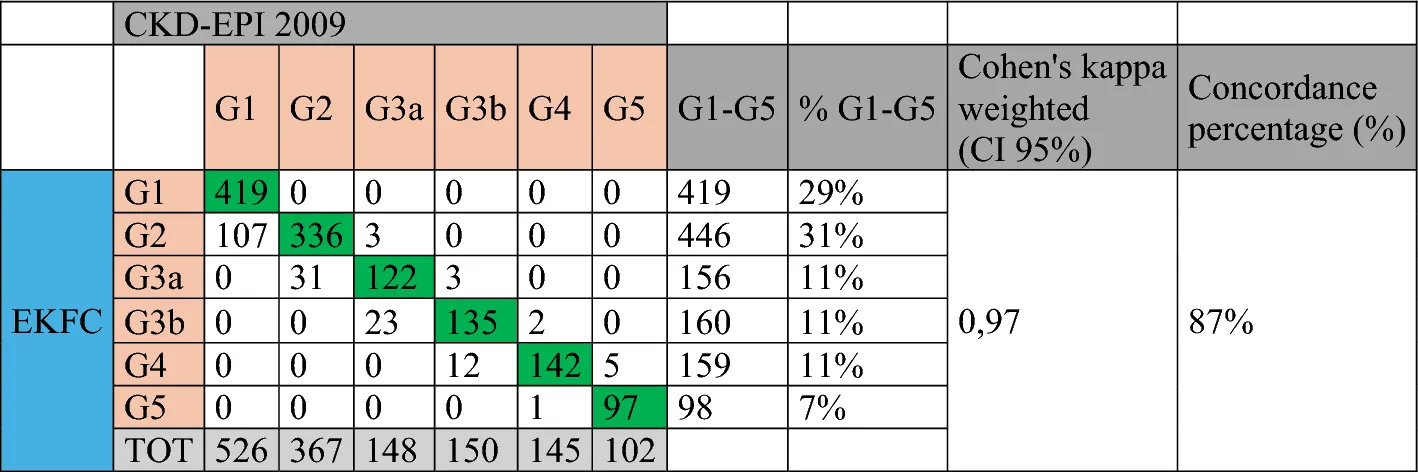
Cross-tabulation between CKD-EPI 2009 and EKFC equations in the CKD staging process

Major reclassifications to more advanced CKD stages can be observed in G1-G3a range, and these are mainly performed by EKFC with respect to CKD-EPI 2009

Overall, these data show that the EKFC equation tends to classify patients into more advanced CKD stages compared to the other equations, highlighting the potential heavy impact of equation selection on clinical CKD staging but keeping consistent with the indications provided by CKD-EPI 2009.

**Fig. 5:**
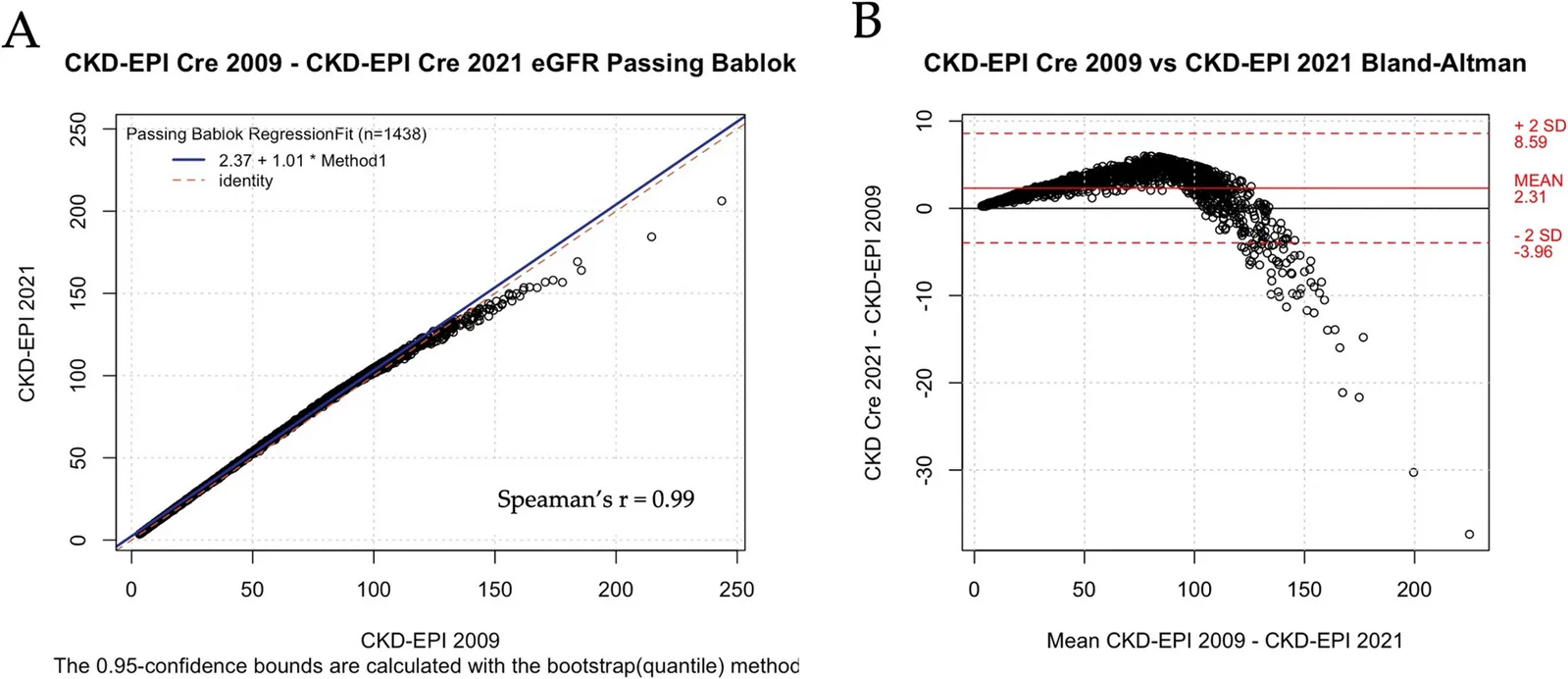
Passing–Bablok and Bland–Altman analysis for comparison between CKD-EPI 2009 and CKD-EPI 2021. **A** Spearman’s correlation coefficient indicates very high concordance (*r* = 0.99) between the two equations as they both share the underlying mathematical infrastructure. **B** Bland–Altman analysis shows a progressively reversing trend as mean values increase

Ultimately, we performed the analytical comparison between creatinine-based CKD-EPI 2009 and CKD-EPI 2021 equation. Figure 5 demonstrates that CKD-EPI 2009 and CKD-EPI 2021 have a very high correlation (*r* = 0.99). The Bland–Altman analysis shows that CKD-EPI 2009 produces progressively higher eGFR values than CKD-EPI 2021 until about 70–80 mL/min/1.73 m^2^, where the trend starts to invert.

Clinically, Table 4 shows that despite the two equations having a very high concordance coefficient (Cohen’s kappa: 0.97), CKD-EPI 2021 reclassified 189 patients to earlier stages. Specifically, 40 out of 148 were downgraded to G2 from G3a and 86 from G2 to G1.

**Table 4:**
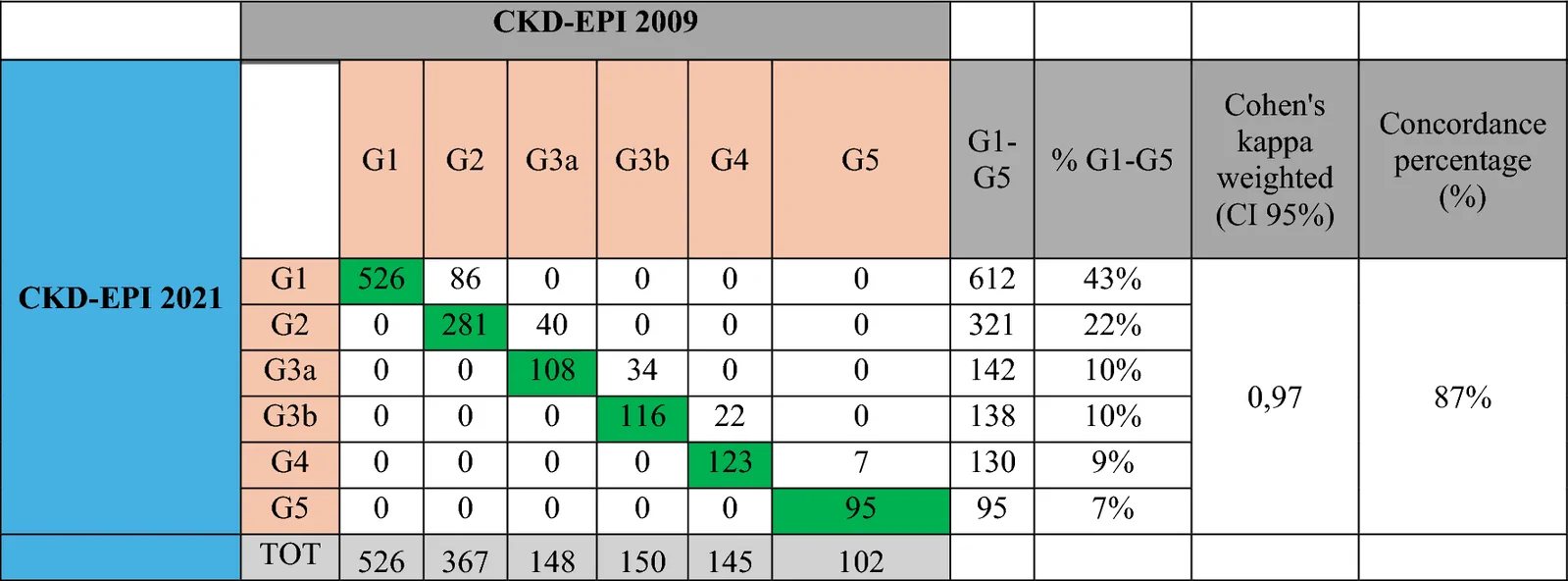
Cross-tabulation between CKD-EPI 2009 and CKD-EPI 2021 equations in the CKD staging process

The two equations within the presented comparison are very similar from a mathematical standpoint. The high Cohen’s weighted kappa is maintained high (0.97), along with an acceptable level of overall concordance (87%), even though 13% of patients were reclassified to earlier CKD stages

Overall these data indicate that the CKD-EPI 2021 systematically produces higher eGFR values than CKD-EPI 2009.

It is well established that serum creatinine may be less reliable in critically ill patients due to factors such as muscle wasting, high comorbidity burden, and fluid resuscitation. Since our study population included 754 patients originating from intensive care units (ICU), we performed additional stratified analyses to account for potential differences in this subgroup.

We first compared mean serum creatinine values between ICU and non-ICU patients using a t-test. ICU patients showed a mean creatinine level of 1.36 ± 1.42 mg/dL, while non-ICU patients showed a mean of 1.56 ± 1.64 mg/dL (*p* = 0.012; *p*-value < 0.05).

Spearman’s correlation coefficients between the selected equations were calculated, and the differences between correlations were assessed using Fisher’s r-to-z transformation. Results are summarized in Table 5.

**Table 5:** Summary of Spearman correlation coefficients deriving from the comparison of the select equations of the determination of creatinine-based eGFR in patients originating from ICU and other departments

Equations	ICU	non-ICU	*p*-value
MDRD vs CKD-EPI 2009	0.97	0.98	0.0015
CKD-EPI vs CKD-EPI 2021	0.99	0.99	0.43
CKD-EPI vs EKFC	0.99	0.99	0.0088

Statistically significant differences between the correlation coefficients were determined through Fisher’s r-to-z transformation

Although statistically significant differences were observed in correlation comparisons between ICU and non-ICU settings, the absolute differences in Spearman coefficients were negligible, suggesting comparable CKD staging behaviors of the compared equations in ICU vs. non-ICU patients.

Consequently, a stratified Bland–Altman analysis was performed. The results confirm what was expected based on the observed differences in mean creatinine levels between ICU and non-ICU patients. In all comparisons, the mean differences were higher in the ICU cohort, indicating that the clinical setting and the patient profile have an impact on GFR estimation. Notably, a marked discrepancy in the mean values of differences was observed for the MDRD-IDMS versus CKD-EPI 2009 comparison between ICU and non-ICU groups: the mean difference was − 17.48 mL/min/1.73 m^2^ in the former and − 4.48 mL/min/1.73 m^2^ in the latter.

## Discussion

In our study, MDRD-IDMS failed to maintain correlation above 80 mL/min/1.73 m^2^. Specifically, our analysis confirmed CKD-EPI 2009 showed stronger correlation with MDRD-IDMS when patients with eGFR values above 120 and 80 mL/min/1.73 m^2^ were excluded. Regarding CKD staging, the two equations are highly correlated and classify patients similarly although CKD-EPI 2009 yields a slightly higher prevalence in stages G3a–G5. These findings may support previous reports [9], suggesting that CKD-EPI 2009 provides a more precise estimation of kidney function in patients with normal or near-normal GFR, with potential implications for CKD diagnosis and epidemiological studies. The EKFC equation is the most recently developed tool for estimating GFR and was derived from data primarily obtained from European cohorts, suggesting that it should be suitable for the population investigated in our study [16]. A recent attempt to replace CKD-EPI equations with EKFC in a northern European hospital, conducted by A. Russel et al. [13] demonstrated that the use of EKFC led to a substantial patient reclassification (11%) toward lower eGFR values, increasing the prevalence of CKD stages G3–G5 from 4.5% to 6.2% [13]. Our findings confirm what is reported in the literature as the EKFC equation reclassified almost 12% of the patients to more advanced stages. Following the EKFC evaluation, we also compared the CKD-EPI 2009 with the CKD-EPI 2021 equation, which was developed to remove race as a factor, aiming to improve CKD detection in Black Americans individuals. EFLM (European Federation of Laboratory Medicine) recommended against the use of such race-correcting factor [11] as current evidence shows that it cannot be applied to population outside the USA and that the use of equations without this factor proved to have better performance [17, 18]. If CKD-EPI 2021 was to be used on our hospital population, there would be a profound change in the CKD prevalence as, while it would allow for a correction of GFR overestimation in Black Americans, it leads to a GFR overestimation from 0.5 a 3.9 mL/min/1,73 m^2^ in White patients. In our predominantly White cohort, CKD-EPI 2021 systematically produced higher eGFR values. In fact, 12.4% of patients were reclassified to earlier CKD stages, particularly from G3a and G2. These findings are in agreement with the EFLM recommendations and mirror what has been previously reported, as CKD-EPI 2021, despite its high correlation with EKFC, CKD-EPI 2009, and MDRD-IDMS, tends to shift White patients toward earlier CKD stages [10, 19]. The choice to switch from MDRD-IDMS to one of the alternative equations must also consider the characteristics of the population to which they will be applied, as the performance of these formulas may vary depending on the cohorts on which they were originally developed. CKD-EPI 2009 was derived from a large, ethnically and clinically heterogeneous adult population (≥ 18 years), including both healthy participants and CKD patients. It demonstrated better agreement with measured GFR than MDRD-IDMS, particularly in individuals who were classified differently. Conversely, the EKFC equation was developed in a European White cohort and was specifically designed to ensure continuity in GFR estimation across pediatric and adult age groups. Although EKFC showed a generally lower bias than CKD-EPI equations recommended by KDIGO, its restricted ethnic representation and pediatric–adult orientation may limit its applicability in certain hospital settings. Our study population included both critically ill and non-critically ill patients, with a large proportion of individuals admitted to the ICU. Serum creatinine and creatinine-based eGFR equations have important limitations in critically ill patients, as acute changes in kidney function, fluid resuscitation, altered volume of distribution, and reduced creatinine generation due to muscle wasting may result in non-steady-state creatinine concentrations, thereby reducing the accuracy of GFR estimation [20, 21]. Our findings showed that ICU patients had significantly lower mean serum creatinine concentrations than non-ICU patients. Although the retrospective design does not allow the underlying causes of this difference to be determined, it is consistent with the effects of fluid resuscitation, altered volume distribution, and other physiological changes commonly observed in critically ill patients. These factors may have introduced a degree of bias in the overall comparison between eGFR equations. Nevertheless, stratified weighted Cohen's kappa analyses yielded results consistent with those observed in the overall cohort, indicating that the agreement patterns between equations remained stable regardless of ICU status. Our study has its limitations. First, it was designed as a preliminary step in the process of replacing the eGFR equation currently used in our laboratory, focusing on the agreement between different estimating equations and the potential consequences for CKD classification. However, further validation studies are needed before any change can be recommended, our study lacks comparison with measured GFR (mGFR), which is essential to determine the accuracy and actual clinical performance of the proposed equations. Second, the study population was clinically heterogeneous, and CKD staging comparisons were performed solely to evaluate the analytical impact of applying different eGFR equations rather than to determine the true prevalence or classification of CKD in specific patient groups. Finally, serum creatinine was measured using two different analytical methods during the study period (kinetic Jaffe before 2018 and an enzymatic assay thereafter). To address this limitation, separate analyses were performed in the two analytical periods, which confirmed the overall analytical agreement and CKD classification concordance between equations (supplementary material). Nevertheless, the retrospective design precluded direct method comparison or calibration studies between the two assays. Therefore, a residual analytical bias related to the change in creatinine measurement methodology cannot be completely excluded.

## Conclusions

In conclusion, our study confirms the MDRD-IDMS’s limited ability to achieve agreement at higher eGFR values and preliminarily supports its replacement with more modern equations. Among the alternatives tested, CKD-EPI 2009 showed the most balanced performance in terms of analytical correlation and CKD staging consistency, whereas EKFC classified a higher number of patients as having reduced kidney function, consistent with previous evidence. Despite the lower bias reported for EKFC in its original development, our hospital predominantly serves an adult population, while pediatric cases are usually referred elsewhere. In addition, our patient base includes ethnic minorities not represented in the EKFC derivation cohort. For these reasons, CKD-EPI 2009 might be a more conservative choice and appropriate equation for our clinical context. Further studies will assess how adopting a different eGFR equation may influence clinical management of patients and its impact on their survival. Moreover, validation against mGFR is essential to determine the accuracy of the proposed equations and to support any future replacement of the currently adopted eGFR equation.

## Supplementary Information

Below is the link to the electronic supplementary material.

Supplementary file1 (DOCX 47 KB)

## Data Availability

Research data statement has not been made available for this paper as it contains sensible information and patient personal data which cannot be shared.
