# Supplementary material for: Retrospective and comparative assessment of MDRD, CKD-EPI, and EKFC equations for estimating glomerular filtration rate in a clinically heterogeneous hospital population
Source: Int Urol Nephrol. 2026 Jul 24;58(12):5677–86. doi: 10.1007/s11255-026-05288-w (PMC13768384; doi:10.1007/s11255-026-05288-w)
Supplement: Supplementary file 1 [file 11255_2026_5288_MOESM1_ESM.docx]

**Supplementary Material: Sensitivity analysis and comparison of results between two**

**study periods in which the Jaffe and enzymatic methods were used, respectively.**

To evaluate the impact of the methodological change between the two time periods on the results showed in the main manuscript, sensitivity analysis and results comparison between these periods was performed. The dataset was split into two subsets which would store eGFR determinations based on Creatinine measurement through Jaffe and enzymatic methods, respectively.

1. **Subsets from original dataset: study population description**

The two subsets from original datasets are composed of different patients with a diversified clinical background. To allow for a better contextualization of the results showed within this supplement, Table S1 describe demographics and ward of origin.

**Table S1.** Summary of the characteristics of the Jaffe and enzymatic subsets. The Jaffe subset included 444 males (58.96%) and 309 females (41.04%) (n = 753), with a mean age of 61.59 years. The enzymatic subset included 442 males (64.53%) and 243 females (35.47%) (n = 685).

| DEMOGRAPHICS | | | | |
| --- | --- | --- | --- | --- |
| JAFFE SUBSET (N = 753) | | | **ENZYMATIC SUBSET (N = 685)** | |
| Age, years (SD) | 61.59 (16.05) | | 61.85 (15.61) | |
|  | **Males** | **Females** | **Males** | **Females** |
| N. patients | 444 | 309 | 442 | 243 |
| Median Scr | 0.9 mg/dL | 0.9 mg/dL | 1.03 mg/dL | 0.95 mg/dL |
| DEPARTMENT | **# Patients** | | **# Patients** | |
| Intensive Care Unit (ICU) | 324 | | 430 | |
| External patients | 275 | | 89 | |
| Surgery and transplants | 92 | | 45 | |
| Cardiac Surgery | 17 | | 25 | |
| Internal Medicine | 19 | | 21 | |
| Gastroenterology | 2 | | 25 | |
| Hepatology | 1 | | 16 | |
| Hypertension and Nephrology | 12 | | 1 | |
| Infectious disease | 5 | | 10 | |
| Stem cells transplant department | 3 | | 8 | |
| Cardiothoracic anaesthesia | 0 | | 9 | |
| Endocrinology and diabetology | 2 | | 7 | |

**2. Spearman’s coefficient**

Spearman’s correlation coefficients were calculated for each one of the equations comparison performed in the main manuscript. Results are shown in Table S2.

| **Equations** | **Jaffe dataset** | **Enzymatic dataset** | **Undivided** |
| --- | --- | --- | --- |
| MDRD-IDMS vs CKD-EPI 2009 | 0.97 | 0.98 | 0.99 |
| CKD-EPI 2009 vs CKD-EPI 2021 | 0.99 | 0.99 | 0.99 |
| CKD-EPI 2009 vs EKFC 2021 | 0.99 | 0.99 | 0.99 |

**Table S2. Spearman’s correlation coefficients comparison**. R coefficients were compared between the selected equations for the eGFR determination.

3. **Bland Altman’s comparisons**

Bland altman’s analysis was performed for each one of the subsets and compared with each other, including the original dataset. Results were summarised in Table S3.

| **MDRD-IDMS vs CKD-EPI 2009** | | | |
| --- | --- | --- | --- |
| **Methods** | **Mean Differences** | **+2SD** | **-2SD** |
| Jaffe | -13.47 | 83.58 | -110.52 |
| Enzymatic | -8.86 | 52.77 | -70.5 |
| Original Dataset | -11.28 | 70.93 | -93.49 |
| **CKD-EPI 2009 vs EKFC 2021** | | | |
| **Methods** | **Mean Differences** | **+2SD** | **-2SD** |
| Jaffe | -5.2 | 6.4 | -16.8 |
| Enzymatic | -4.57 | 5.17 | -14.3 |
| Original Dataset | -4.9 | 5.87 | -15.67 |
| **CKD-EPI 2009 vs CKD-EPI 2021** | | | |
| **Methods** | **Mean Differences** | **+2SD** | **-2SD** |
| Jaffe | 2.2 | 9.35 | -4.96 |
| Enzymatic | 2.44 | 7.57 | -2.69 |
| Original Dataset | 2.31 | 8.59 | -3.96 |

**Table S3 Summary table of parameters obtained through Bland Altman’s analyses between equations.** Mean differences, +2SD and -2SD are reported to show differences between utilized methods for creatinine determination.

These results show that mean differences are lower in the enzymatic subset than in the Jaffe subset for two of the three comparisons. For CKD-EPI 2009 vs CKD-EPI 2021, the mean difference is slightly higher in the enzymatic subset, although it remains comparable to that observed in the Jaffe subset.

4. **CKD Staging**

CKD staging performance of the selected eGFR equations was evaluated in the Jaffe and enzymatic subsets to confirm findings obtained on the whole population. The confusion matrices were grouped up in Table S4.

|  |  | **CKD-EPI 2009** | | | | | |  |  |  |  |  |  |  |  |  |  |  |  |
| --- | --- | --- | --- | --- | --- | --- | --- | --- | --- | --- | --- | --- | --- | --- | --- | --- | --- | --- | --- |
|  |  |  |  |  |  |  |  |  |  |  |  |  |  |  |  |  |  |  |  |
| **MDRD-IDMS** |  | **G1** | **G2** | **G3a** | **G3b** | **G4** | **G5** | **TOTAL** | **%** |  |  | **G1** | **G2** | **G3a** | **G3b** | **G4** | **G5** | **TOTAL** | **%** |
|  | **G1** | 259 | 27 | 0 | 0 | 0 | 0 | 286 | 38% |  | **G1** | 215 | 15 | 0 | 0 | 0 | 0 | 230 | 31% |
|  | **G2** | 28 | 161 | 2 | 0 | 0 | 0 | 191 | 25% |  | **G2** | 24 | 144 | 1 | 0 | 0 | 0 | 169 | 22% |
|  | **G3a** | 0 | 10 | 63 | 4 | 0 | 0 | 77 | 10% |  | **G3a** | 0 | 10 | 75 | 6 | 0 | 0 | 91 | 12% |
|  | **G3b** | 0 | 0 | 1 | 74 | 6 | 0 | 81 | 11% |  | **G3b** | 0 | 0 | 6 | 60 | 0 | 0 | 66 | 9% |
|  | **G4** | 0 | 0 | 0 | 3 | 66 | 1 | 70 | 9% |  | **G4** | 0 | 0 | 0 | 3 | 73 | 4 | 80 | 11% |
|  | **G5** | 0 | 0 | 0 | 0 | 0 | 48 | 48 | 6% |  | **G5** | 0 | 0 | 0 | 0 | 0 | 49 | 49 | 7% |
|  | **TOTAL** | 287 | 198 | 66 | 81 | 72 | 49 | 753 |  |  | **TOTAL** | 239 | 169 | 82 | 69 | 73 | 53 | 685 |  |
|  | **%** | 38% | 26% | 9% | 11% | 10% | 7% |  |  |  |  | 32% | 22% | 11% | 9% | 10% | 7% |  |  |
|  |  |  |  |  |  |  |  |  |  |  |  |  |  |  |  |  |  |  |  |
|  |  | **CKD-EPI 2021** | | | | | |  |  |  |  |  |  |  |  |  |  |  |  |
|  |  |  |  |  |  |  |  |  |  |  |  |  |  |  |  |  |  |  |  |
| **CKD-EPI 2009** |  | **G1** | **G2** | **G3a** | **G3b** | **G4** | **G5** | **TOTAL** | **%** |  |  | **G1** | **G2** | **G3a** | **G3b** | **G4** | **G5** | **TOTAL** | **%** |
|  | **G1** | 287 | 0 | 0 | 0 | 0 | 0 | 287 | 38% |  | **G1** | 239 | 0 | 0 | 0 | 0 | 0 | 239 | 32% |
|  | **G2** | 49 | 149 | 0 | 0 | 0 | 0 | 198 | 26% |  | **G2** | 37 | 132 | 0 | 0 | 0 | 0 | 169 | 22% |
|  | **G3a** | 0 | 17 | 49 | 0 | 0 | 0 | 66 | 9% |  | **G3a** | 0 | 23 | 59 | 0 | 0 | 0 | 82 | 11% |
|  | **G3b** | 0 | 0 | 21 | 60 | 0 | 0 | 81 | 11% |  | **G3b** | 0 | 0 | 13 | 56 | 0 | 0 | 69 | 9% |
|  | **G4** | 0 | 0 | 0 | 14 | 58 | 0 | 72 | 10% |  | **G4** | 0 | 0 | 0 | 8 | 65 | 0 | 73 | 10% |
|  | **G5** | 0 | 0 | 0 | 0 | 2 | 47 | 49 | 7% |  | **G5** | 0 | 0 | 0 | 0 | 5 | 48 | 53 | 7% |
|  | **TOTAL** | 336 | 166 | 70 | 74 | 60 | 47 | 753 |  |  | **TOTAL** | 276 | 155 | 72 | 64 | 70 | 48 | **685** |  |
|  | **%** | 45% | 22% | 9% | 10% | 8% | 6% |  |  |  |  | 37% | 21% | 10% | 8% | 9% | 6% |  |  |
|  |  |  |  |  |  |  |  |  |  |  |  |  |  |  |  |  |  |  |  |
|  |  | **EKFC** | | | | | |  |  |  |  |  |  |  |  |  |  |  |  |
|  |  |  |  |  |  |  |  |  |  |  |  |  |  |  |  |  |  |  |  |
| **CKD-EPI 2009** |  | **G1** | **G2** | **G3a** | **G3b** | **G4** | **G5** | **TOTAL** | **%** |  |  | **G1** | **G2** | **G3a** | **G3b** | **G4** | **G5** | **TOTAL** | **%** |
|  | **G1** | 237 | 50 | 0 | 0 | 0 | 0 | 287 | 38% |  | **G1** | 182 | 57 | 0 | 0 | 0 | 0 | 239 | 32% |
|  | **G2** | 0 | 183 | 15 | 0 | 0 | 0 | 198 | 26% |  | **G2** | 0 | 153 | 16 | 0 | 0 | 0 | 169 | 22% |
|  | **G3a** | 0 | 1 | 52 | 13 | 0 | 0 | 66 | 9% |  | **G3a** | 0 | 2 | 70 | 10 | 0 | 0 | 82 | 11% |
|  | **G3b** | 0 | 0 | 1 | 71 | 9 | 0 | 81 | 11% |  | **G3b** | 0 | 0 | 2 | 64 | 3 | 0 | 69 | 9% |
|  | **G4** | 0 | 0 | 0 | 1 | 70 | 1 | 72 | 10% |  | **G4** | 0 | 0 | 0 | 1 | 72 | 0 | 73 | 10% |
|  | **G5** | 0 | 0 | 0 | 0 | 2 | 47 | 49 | 7% |  | **G5** | 0 | 0 | 0 | 0 | 3 | 50 | 53 | 7% |
|  | **%** | 237 | 234 | 68 | 85 | 81 | 48 | 753 |  |  | **TOTAL** | 182 | 212 | 88 | 75 | 78 | 50 | 685 |  |
|  |  | 31% | 31% | 9% | 11% | 11% | 6% |  |  |  | **%** | 24% | 28% | 12% | 10% | 10% | 7% |  |  |

**Table S4. Direct comparison of CKD staging according to the selected eGFR equations.** Comparisons performed in the Jaffe subset are presented in the left column, whereas those performed in the enzymatic subset are shown in the right column. Percentages are expressed relative to the total number of patients in each respective subset.

Furthermore, Cohen’s kappa for each comparison between equations on CKD staging was calculated and compared with the ones obtained on the original dataset. Table S5 shows negligible to no difference between the coefficients, indicating a high level of concordance and confirmation of the findings originating from analysis performed on the original dataset.

| **Equations** | **Jaffe subset** | **Enzymatic subset** | **Original dataset** |
| --- | --- | --- | --- |
| MDRD-IDMS vs CKD-EPI 2009 | 0.98 | 0.98 | 0.98 |
| CKD-EPI 2009 vs CKD-EPI 2021 | 0.98 | 0.98 | 0.98 |
| CKD-EPI 2009 vs EKFC 2021 | 0.98 | 0.97 | 0.97 |

**Table S5. Summary of Cohen’s kappa.** Table shows the Cohen’s kappa which were recalculated on both Jaffe and Enzymatic subset, showing no relevant differences between levels of concordance from the original dataset.

*4.1 MDRD-IDMS vs CKD-EPI 2009*

CKD classification performed in both the Jaffe and enzymatic subsets confirmed the main findings observed in the overall cohort. In the Jaffe subset, 5.6% of patients were reclassified by CKD-EPI 2009 into earlier stages (42/753), including 28 from G2 to G1, 10 from G3a to G2, 1 from G3b to G3a, and 3 from G4 to G3b. A similar pattern was observed in the enzymatic subset, where 6.3% of patients (43/685) were reclassified to earlier stages, including 24 from G2 to G1, 10 from G3a to G2, 6 from G3b to G3a, and 3 from G4 to G3b.

Overall, these findings confirm that the CKD-EPI 2009 equation tends to yield higher estimated GFR values compared with the MDRD-IDMS equation, resulting in a systematic shift toward less severe CKD stages. Table S3 shows that concordance (Cohen’s kappa) is identical between the subsets.

*4.2 CKD-EPI 2009 vs CKD-EPI 2021*

CKD-EPI 2021 reclassified 103 (13%) and 86 (12.5%) patients to earlier stages in the Jaffe and enzymatic subsets, respectively. Particular attention should be paid to the G2-G3 patient population, for which CKD-EPI 2021 reclassified 17/753 (2.25%) patients in the Jaffe subset and 23/685 (3.35%) patients in the enzymatic subset, from G3a to G2. Overall, in both the Jaffe and enzymatic subsets, CKD-EPI 2021 systematically produced higher eGFR values than CKD-EPI 2009, reclassifying large groups of patients to earlier stages as expected. These findings confirmed what was found on the original dataset.

*4.3 CKD-EPI 2009 vs EKFC*

In the first subset, EKFC reclassified 88/753 patients (11.68%) to more advanced CKD stages. Specifically, 50 patients from G1 to G2 and 15 from G2 to G3a.
In the enzymatic subset, 86/685 (12.5%) were assigned by EKFC to more advanced stages, with 57 patients from G1 to G2 and 16 patients from G2 to G3a. Overall these results confirm the EKFC tendency to assign patients to more advanced stages than CKD-EPI 2009.
